# *Pseudomonas shiyinii* ST4 inhibits sexual mating of *Sporisorium scitamineum* through the GacS/GacA two-component system and the *mib1* gene cluster

**DOI:** 10.1128/aem.00321-26

**Published:** 2026-09-15

**Authors:** Yihang Yang, Nuoqiao Lin, Jiahui Huang, Weihan Gu, Zhijian Hu, Kai Yin, Xinping Bi, Ling He, Lian-Hui Zhang, Zhibin Liang, Yizhen Deng

**Affiliations:** 1 Guangdong Provincial Key Laboratory of Microbial Signals and Disease Control, Integrative Microbiology Research Center, South China Agricultural University 578635, Guangzhou, China; 2 State Key Laboratory of Biocontrol, School of Life Sciences, Sun Yat-Sen University 26469, Guangzhou, China; 3 Hubei Key Laboratory of Edible Wild Plants Conservation and Utilization, College of Life Sciences, Hubei Normal University 98425, Huangshi, China; Michigan State University, East Lansing, Michigan, USA

**Keywords:** sugarcane smut disease, MibR1, MibR2, RsmY, CsrA

## Abstract

Sugarcane smut is one of the most devastating fungal diseases affecting sugarcane plantations. The virulence of the causative agent, *Sporisorium scitamineum*, depends on a process called sexual mating of compatible sporidia MAT-1 and MAT-2 to form infectious dikaryotic hyphae. We previously isolated *Pseudomonas shiyinii* strain ST4, capable of inhibiting the fungal sexual mating. However, its biocontrol mechanism remains unclear. In this study, Tn5 mutagenesis of strain ST4 led to the identification of a number of genes potentially associated with the biosynthesis of mating inhibitors. Among them, a two-component system GacS/GacA was found responsible for the inhibitory activity against fungal mating. Transcriptomic and genetic analyses unveiled that GacS/GacA positively regulates a novel gene cluster designated as *mib1* (mating inhibitor biosynthesis 1), consisting of seven genes. Compared with the parental strain ST4, the *mib1* deletion mutants were much compromised in the anti-mating activity. Two LysR-family transcriptional regulators, located adjacent to the *mib1* gene cluster, were also shown to play key roles in regulating *mib1* transcription and biosynthesis of mating inhibitor(s). In summary, the results from this study uncovered a novel gene cluster, the two-component system GacS/GacA, and two transcriptional factors associated with the regulation and biosynthesis of inhibitory compound(s) against *S. scitamineum* sexual mating. The findings depict the genetic regulatory network that controls the biocontrol potency of *Pseudomonas shiyinii* ST4 and present useful clues for identification of its anti-mating inhibitor(s).

**IMPORTANCE:**

*Sporisorium scitamineum* is the major sugarcane pathogen that causes tremendous economic losses internationally. The sporidia sexual mating is vital for the fungus to form dikaryotic hyphae capable of infecting sugarcanes, thus is a highly attractive and promising target for screening of biocontrol agents and green chemical mating inhibitors against this devastating fungal pathogen. We previously reported that *Pseudomonas shiyinii* ST4 can inhibit the sexual mating of *S. scitamineum* without affecting its haploid sporidia growth. By using transposon mutagenesis in combination with genetic and bioinformatics analyses, this study aimed to identify the genetic components of strain ST4 associated with its biocontrol potency. The significance of this study is in (i) identifying a novel gene cluster *mib1* involved in production of mating inhibitor(s) by strain ST4, (ii) demonstrating that the GacS/GacA-RsmY-CsrA regulatory cascade plays an essential role in regulation of the *mib1* cluster and the bacterial anti-mating activity, and (iii) unveiling two novel transcription factors, that is, MibR1 and MibR2, which act downstream of the Gac regulatory cascade in modulation of the *mib1* gene expression. The findings represent a critical step in depicting the biocontrol mechanisms of strain ST4 and provide valuable clues for further investigations.

## INTRODUCTION

Sugarcane is widely planted in tropical and subtropical regions and is rich in sugar, water, and other substances, making it an important economic crop (1). Sugarcane smut disease is the most severe fungal disease affecting sugarcane plantations (2). The causal agent, *Sporisorium scitamineum*, has an interesting life cycle comprising three phases: the yeast-like haploid sporidia phase, the dikaryotic hyphae phase, and the diploid teliospore phase (3, 4). The yeast-like haploid sporidia occur in two mating types, MAT-1 and MAT-2; neither is pathogenic, and both are incapable of infecting host plants. Following sexual mating between compatible mating types, dikaryotic hyphae are generated that possess full virulence and can invade host plants (5). The dikaryotic hyphae grow upward to sugarcane apex, penetrate the young buds of sugarcane to develop into dark brown–black teliospore masses, the most recognized symptom known as “black whip” (6). The “black whip” is densely covered with millions of teliospores, which are diploid. Under suitable conditions, these teliospores germinate and undergo meiosis to produce haploid cells of MAT-1 and MAT-2 (7). Thus, sexual mating and subsequent dikaryotic hyphae generation constitute the critical steps enabling *S. scitamineum* to systemically infect sugarcane. Recent studies indicate that the sexual mating and dikaryotic hyphae growth of *Sporisorium scitamineum* are associated with the a and b loci, the transcription factor Prf1, intracellular G proteins, and the cAMP/MAPK signaling cascade (3, 5, 8, 9). Control of sugarcane smut disease remains to be a great challenge (10–12). Chemical pesticides face the difficulties of penetrating sugarcane's tough and waxy surface, as well as the problem of environmental contamination. Breeding resistant varieties demands a long breeding cycle and an inclination to lose resistance to the newly emerged physiological races of *S. scitamineum* (13).

We recently isolated a rhizospheric *Pseudomonas shiyinii* strain, named ST4 (txid:1856588), that specifically suppresses sexual mating and dikaryotic hyphae growth, whereas generating hardly any effect on haploid sporidia growth of *S. scitamineum* (14, 15). In addition, strain ST4 could also inhibit the sexual mating and the virulence of *Ustilago maydis* (16, 17), indicating a promising biocontrol potential against other smut fungal diseases. Strain ST4 is most similar to *Pseudomonas guariconensis* (txid:1288410) (18), but contains genetic variations (15). By introducing a sugar metabolic gene, an engineered strain ST4E showed an enhanced ability to colonize and proliferate in sugarcane and inhibit the fungal sexual mating (16). However, it remains unidentified which genes encode the sexual mating inhibitor(s) and the associated regulatory mechanisms. In this study, a novel gene cluster responsible for biosynthesis of *S. scitamineum* mating inhibitory compound(s) and under the regulation of the two-component system GacS/GacA was identified by Tn5 transposon insertion mutation, genetic analysis, and transcriptome data analysis. In addition, two transcriptional regulatory genes associated with the regulation of mating inhibitor(s) production were also identified. These findings pave the way to elucidating the biocontrol mechanisms of *Pseudomonas shiyinii* ST4 against the sugarcane smut disease caused by *S. scitamineum*.

## RESULTS

### GacS/GacA two-component system is essential for the inhibitory activity of strain ST4 against *S. scitamineum* sexual mating

**Fig 1:**
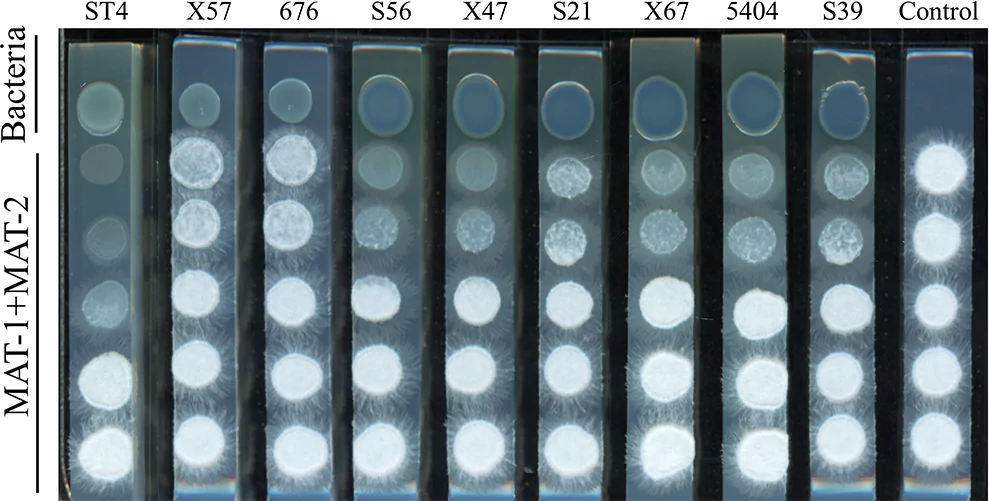
Transposon insertional mutants of *Pseudomonas shiyinii* strain ST4 with a defect in inhibiting *S. scitamineum* sexual mating. The experiments were done independently at least three times, and the representative images are shown here. Control: the fungal cells alone without strain ST4 or its derivative.

In order to understand the biocontrol mechanism of *Pseudomonas shiyinii* ST4, we used a Tn5 transposon library to screen for mutants that could not inhibit the sexual mating of *S. scitamineum*. Among the total of over 40,000 Tn5 mutants, eight strains were found to have lost or partially attenuated inhibitory activity (Fig. 1). High-efficiency Thermal Asymmetric Interlaced PCR (hiTAIL-PCR) and sequence analysis were then conducted to identify the Tn5-inserted genes in strain ST4 responsible for inhibiting sexual mating. The results showed that *gacS* and *gacA* were disrupted by Tn5 in the anti-mating deficient mutants, while in the partially attenuated mutants, the *gcd* gene and the genes of the *pqq* cluster were tagged by Tn5 (Table 1).

**TABLE 1:** *Pseudomonas shiyinii* ST4 mutants defective in inhibition of *S. scitamineum* sexual mating

Strain	Gene ID	Gene	Predicted product
X57	tmp_000901	*gacS*	Sensor kinase
676	tmp_001608	*gacA*	UvrY/SirA/GacA family response regulator transcription factor
S56	tmp_000774	*gcd*	Glucose/quinate/shikimate family membrane-bound PQQ-dependent dehydrogenase
X47	tmp_000121	*pqqB*	Pyrroloquinoline quinone biosynthesis protein
S21	tmp_000120	*pqqC*	Pyrroloquinoline quinone synthase
X67	tmp_000119	*pqqD*	Pyrroloquinoline quinone biosynthesis peptide chaperone
5404	tmp_000118	*pqqE*	Pyrroloquinoline quinone biosynthesis protein
S39	tmp_000123	*pqqF*	Pyrroloquinoline quinone biosynthesis protein

Among these eight genes, five genes (*pqqBCDEF*) are involved in the biosynthesis of pyrroloquinoline, which is a common cofactor of many metabolic enzymes (19). The *gcd* gene encodes a putative glucose/quinate/shikimate family membrane-bound PQQ-dependent dehydrogenase. The remaining two genes, *gacS* and *gacA*, encode a two-component system, which share about 78% and 92% similarity at the amino acid level, respectively, with the well-characterized counterparts in *Pseudomonas aeruginosa* PAO1 (20). The above results suggest that the biosynthesis of *S. scitamineum* mating inhibitor(s) in strain ST4 may involve enzymes with pyrroloquinoline as a cofactor and might be under the control of the GacS/GacA two-component system.

**Fig 2:**
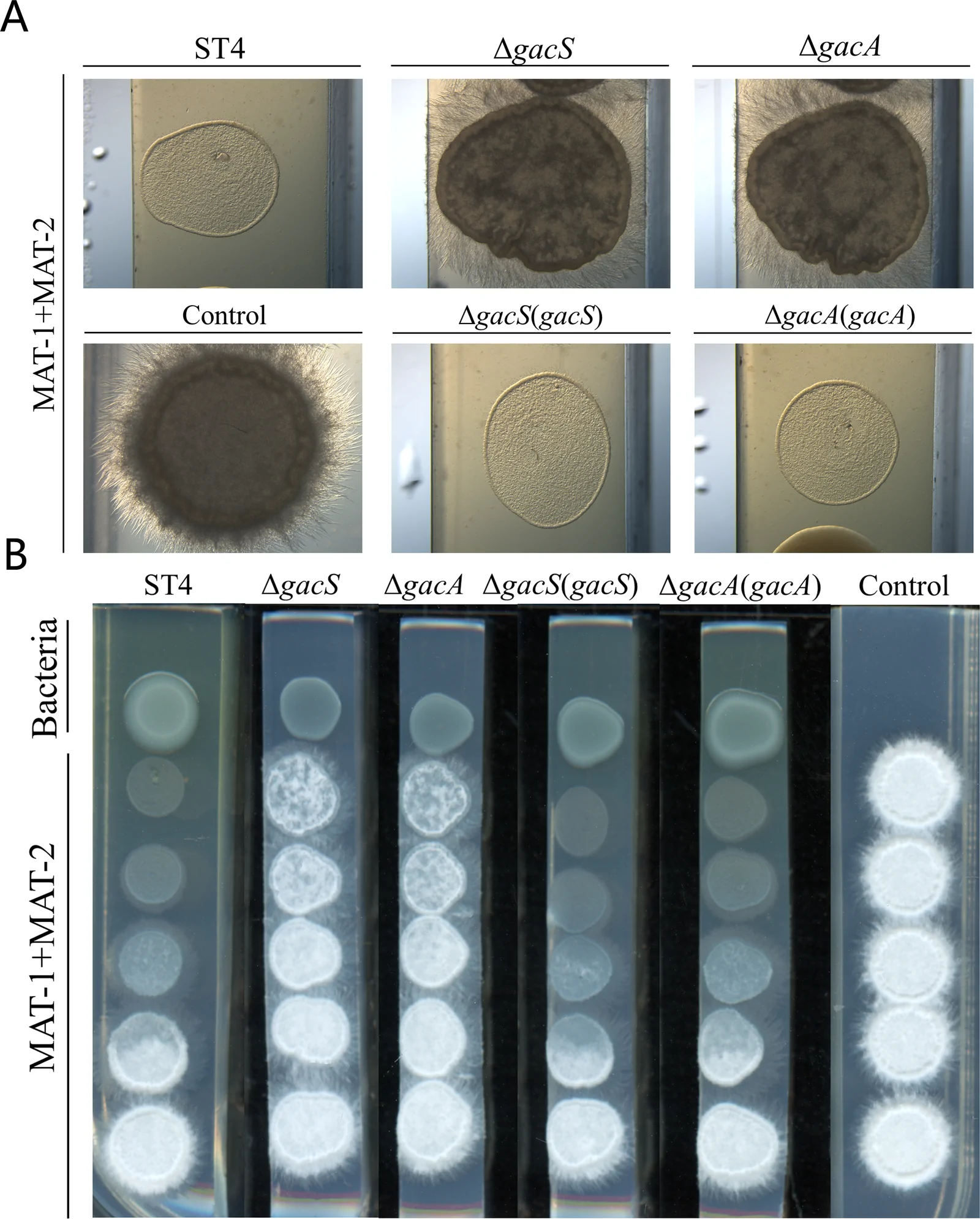
GacS/GacA two-component system is required for inhibition of *S. scitamineum* sexual mating. (**A**) Colony morphology of *S. scitamineum* next to strain ST4. (**B**) Bioassay of the anti-mating activity of the *gacS* and *gacA* deletion mutants and corresponding complemented strains. The experiments were done independently at least three times, and the representative images are shown here. Control: the fungal cells alone without strain ST4 or its derivative.

The GacS/GacA two-component system consists of the sensor kinase GacS and the response regulator GacA, which are widely conserved in various gram-negative bacterial genera, such as *Pseudomonas*, *Salmonella*, and *Legionella*, regulating the production of secondary metabolites and/or extracellular enzymes, and bacterial ecological fitness or tolerance (20, 21). However, the biological function of GacS/GacA has not yet been identified in strain ST4 or its most similar species *P. guariconensis*. To validate whether *gacS* and *gacA* control the biocontrol function of strain ST4, we constructed the *gacS* and *gacA* deletion mutants and corresponding complemented strains, respectively, and assayed against *S. scitamineum* sexual mating. The results showed that the inhibitory activity of mutants was almost completely abolished in Δ*gacS* and Δ*gacA*, which was consistent with the phenotype of the corresponding Tn5 insertion mutants. The mating inhibitory activity was fully restored to the wild-type level in the complemented strains (Fig. 2). Overall, this result confirms that the GacS/GacA two-component system is responsible for the inhibition of *S. scitamineum* sexual mating.

### Identification of the genes potentially under GacS/GacA regulation

**Fig 3:**
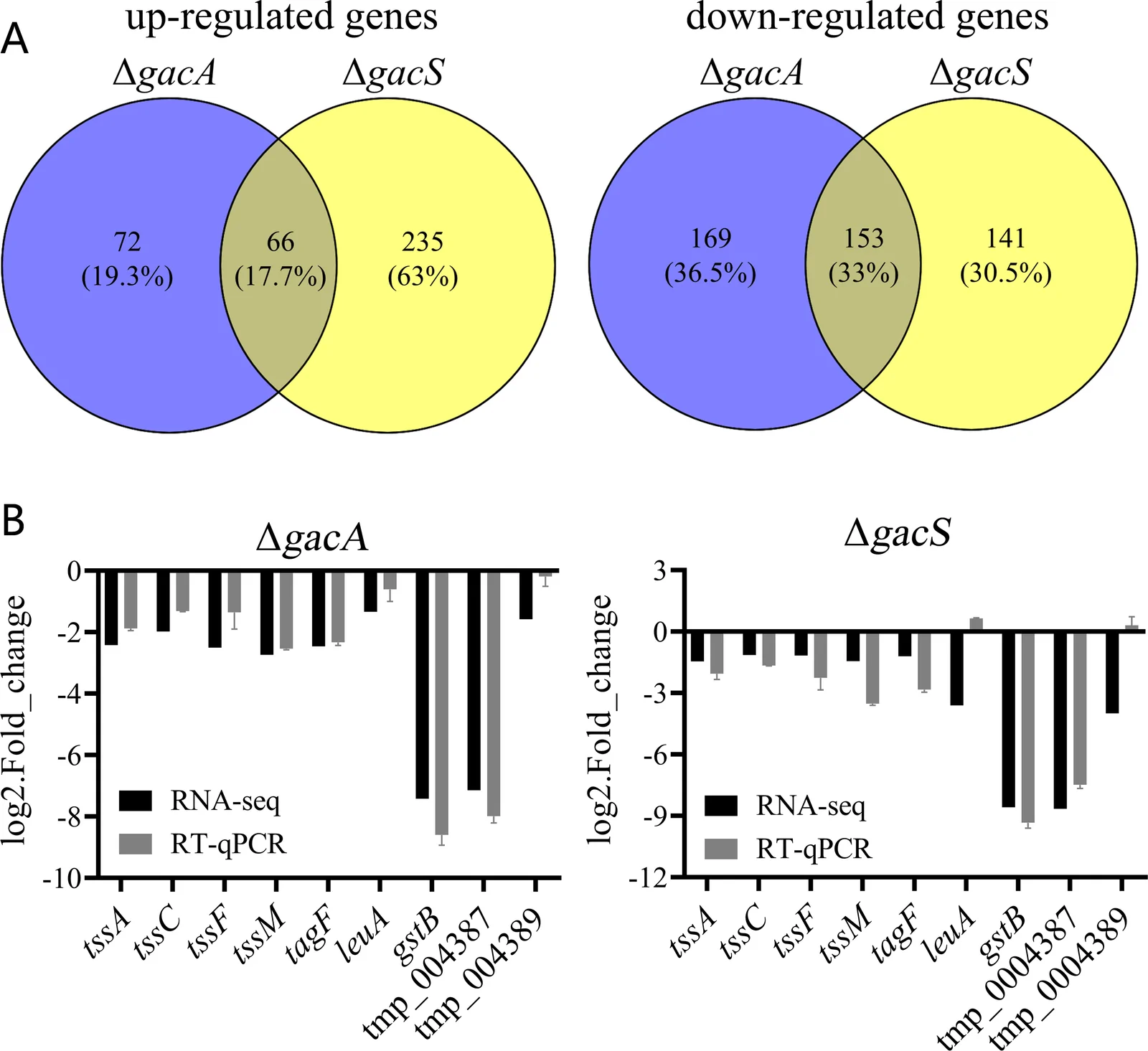
Differentially expressed genes between wild-type ST4 and *gac* mutants. (**A**) Number of upregulated (left) and number of downregulated genes (right) in the *gacS* and *gacA* deletion mutants compared to wild-type strain ST4, respectively. Overlap areas represent the genes co-regulated by GacS and GacA. (**B**) RT-qPCR validation of the mutant Δ*gacA* (left) and mutant Δ*gacS* (right) RNA-seq data with seven randomly selected genes. The RNA extraction method used for RT-qPCR is consistent with that used for RNA-seq. The experiments were done independently at least three times, and the representative images are shown here.

To identify the genes under the control of GacS/GacA, we conducted transcriptome analysis of wild-type ST4 and its mutants Δ*gacS* and Δ*gacA,* respectively. The data showed that, compared with strain ST4, there were 595 genes differently expressed (|log2FC| ≥ 1) in mutant Δ*gacS*, including 301 genes significantly upregulated and 294 genes significantly downregulated. In mutant Δ*gacA*, 460 genes exhibited a significant difference in transcript levels, comprising 138 upregulated and 322 downregulated genes. Among them, 219 genes were co-regulated by GacS and GacA, including 66 upregulated and 153 downregulated (Tables S1 and S2; Fig. 3A). The most highly upregulated (log2FC > 2) common genes include those encoding FAD-binding oxidoreductase, ABC transporter permease, and sodium/proline symporter PutP (Table S3), and the most highly downregulated (log2FC < −2) common genes include those encoding aminotransferase class I/II-fold pyridoxal phosphate-dependent enzyme, glutathione S-transferase family protein, and multidrug efflux MFS transporter (Table S4). The RNA-seq results have been validated by RT-qPCR analyses for seven randomly chosen genes. The results confirmed that expression patterns of these seven genes in the mutants Δ*gacA* and Δ*gacS* were highly consistent with the RNA-seq data (Fig. 3B).

Biocontrol bacteria commonly produce one or more compounds to inhibit the growth or pathogenic processes of corresponding pathogens. These bioactive compounds are typically synthesized by a series of enzymes expressed from different gene clusters (22). We then searched for the gene clusters positively regulated by the GacS/GacA system through systemic examination of the RNA-seq data. Seven gene clusters co-regulated by GacS and GacA were found (Table S5). Among them, only gene clusters 1 and 6 have been characterized, whereas the biological functions of gene clusters 2–5 and 7 have not yet been reported. Cluster 1 encodes components of the widely conserved type VI secretion system (T6SS) and plays a role in niche competition against other bacterial species (23, 24). Cluster 6 has been identified and characterized in *Pseudomonas stutzeri* (25), *Rhodobacter sphaeroides* (26), and *Rhodobacter capsulatus* (27), and is known for encoding the cbb₃-type cytochrome c oxidases, which are key components of bacterial aerobic respiration (28). Overall, by comparative transcriptome analysis, we identified differentially expressed genes common in Δ*gacS* or Δ*gacA* mutants compared to ST4. Among these, we further identified the gene cluster potentially responsible for biosynthesis of mating inhibitor(s) under the regulation of the GacS/GacA two-component system.

### Characterization of a gene cluster and its two transcriptional regulators essential for the anti-mating effect of strain ST4

**Fig 4:**
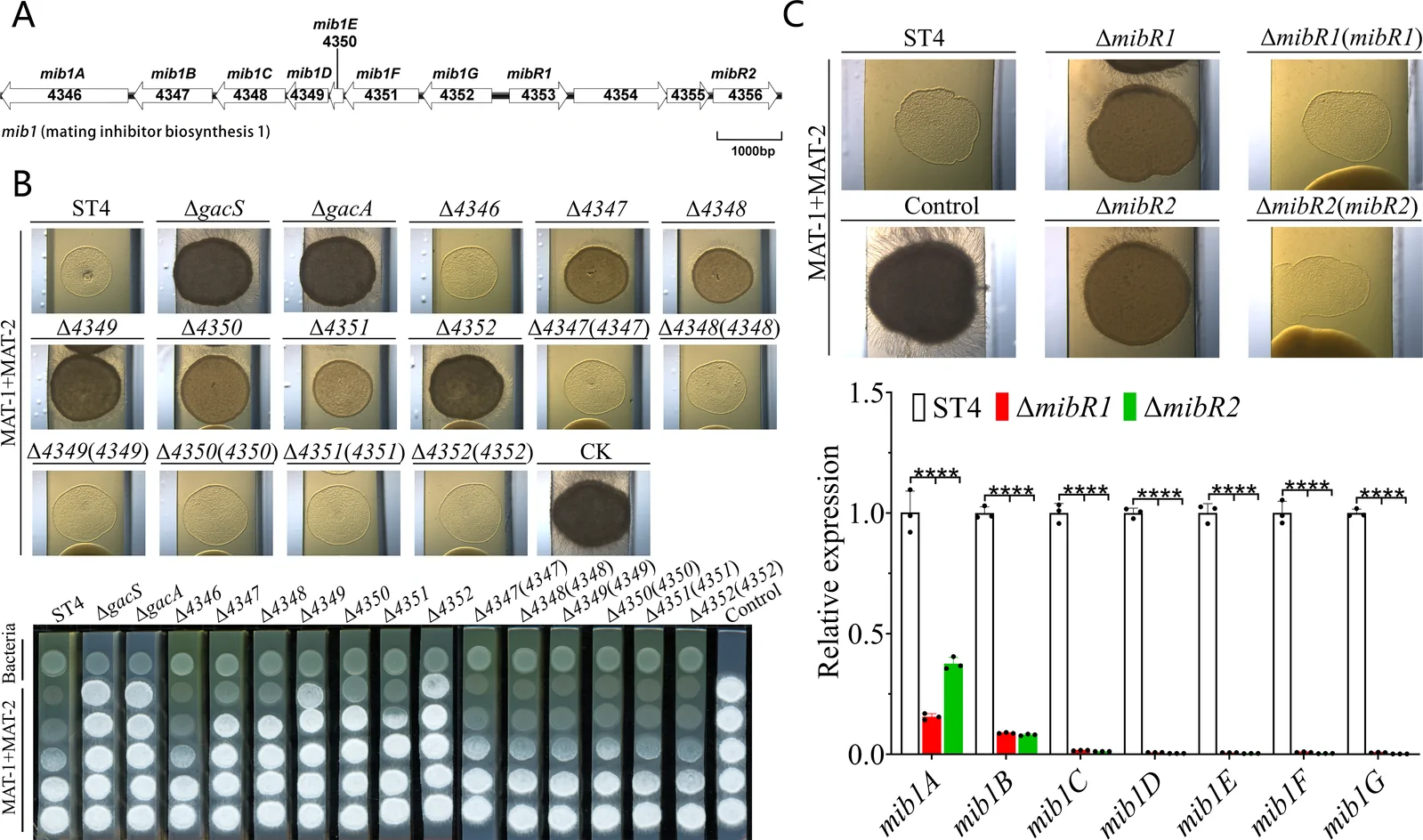
The *mib1* gene cluster and its two transcriptional regulators are essential for the anti-mating activity of strain ST4. (**A**) Genetic map of the *mib1* gene cluster. (**B**) The top panel shows the colony morphology of *S. scitamineum* next to strain ST4. The bottom panel shows the effects of gene deletion and complementation in the *mib1* gene cluster on the ability of ST4 to inhibit sexual mating. (**C**) The top panel shows the colony morphology of *S. scitamineum* next to strain ST4. The bottom panel shows the relative expression of the *mib1* genes in strain ST4, mutants Δ*mibR1,* and Δ*mibR2*, measured by RT-qPCR. Control: the fungal cells alone without strain ST4 or its derivative. All experiments were performed independently at least three times, and representative images are shown here. Statistical analysis was performed using two-way ANOVA with multiple comparisons, and significance with respect to strain ST4 is indicated as ****, *P* < 0.0001. Error bars indicate the standard error of the mean (*n* = 3).

Among the seven gene clusters that are co-regulated by GacS and GacA, we found that all the genes within gene cluster 7 were significantly downregulated, and the fold change of downregulation was relatively larger than the genes in other gene clusters (Fig. S1, Table S5). Therefore, we set out to determine whether gene cluster 7 is associated with the biocontrol function of strain ST4. To this end, we knocked out each gene within cluster 7 and individually constructed the corresponding complemented strains. The results showed that, except for gene *4346*, mutation of genes *4347–4352* reduced the ability of strain ST4 to inhibit the sexual mating of *S. scitamineum*. Particularly, in-frame deletion of genes *4349* and *4352* almost abolished the inhibitory activity against the sexual mating of *S. scitamineum* in a way similar to the Δ*gacS* and Δ*gacA* mutants (Fig. 4B). To investigate whether the *4346-4352* genes are transcribed as a single transcriptional unit, we analyzed the intergenic regions of open reading frames (ORFs) by reverse transcription PCR (RT-PCR). As shown in Fig. S2, gene cluster 7 is transcribed as a single operon. We propose that this gene cluster is a biosynthetic gene cluster responsible for the production of the biocontrol compound(s) in strain ST4, and hereby designated it as the *mib1* (mating inhibitor biosynthesis 1) gene cluster (Fig. 4A). Therefore, genes *4346–4352* were named *mib1A*, *mib1B*, *mib1C*, *mib1D*, *mib1E*, *mib1F*, and *mib1G*, respectively. Among them, *mib1A* encodes 3-isopropylmalate dehydratase; *mib1B*, a multidrug efflux MFS transporter (29, 30); *mib1C*, a cyclase family protein (31); *mib1D*, a glutathione S-transferase family protein (32, 33); *mib1E*, a 4-oxalocrotonate tautomerase family protein (34); *mib1F*, an aminotransferase class I/II-fold pyridoxal phosphate-dependent enzyme (35); and *mib1G*, an iron-containing redox enzyme family protein (36) (Table S5).

Homology searches revealed that the homologues of the *mib1* gene cluster were absent in the representative model strains of the *Pseudomonas* genus, including *P. aeruginosa* PAO1, *P. fluorescens* CHA0, and *P. putida* KT2440. In contrast, a *mib1* cluster homolog was detected in *P. guariconensis* 20-MO00619-0, a strain closely related to strain ST4, exhibiting a high nucleotide sequence identity of about 93%. Additionally, homologous regions similar to the *mib1* gene cluster with relatively high sequence identity were identified in *P. mediterranea* MRN1 and *Pseudomonas* sp. B21-028, both sharing about 73% sequence identity with the *mib1* gene cluster of ST4. Furthermore, homologous regions with notable sequence identity were also detected in several non-*Pseudomonas* species, including *Burkholderia plantarii* ATCC 43733 (~74% identity), *Cupriavidus gilardii* J11 (~72.% identity), and *Paraburkholderia antibiotica* G-4-1-8 (~70% identity) (Fig. S3).

In the vicinity of the *mib1* gene cluster, we noted that two genes, *4353* and *4356,* encode putative LysR-family transcriptional regulators. Bioinformatics analysis showed that they both contain two conserved domains, that is, an N-terminal DNA-binding domain and a C-terminal ligand-binding domain. The N-terminal DNA-binding domain contains a helix-turn-helix (HTH) structural motif, putatively associated with DNA binding, while the C-terminal ligand-binding domain may be involved in sensing signal inducers and determining regulatory specificity (Fig. S4). To verify whether these two regulatory genes play a role in regulating the expression of the *mib1* gene cluster, we generated deletion mutants of these two genes individually. RT-qPCR verified that the transcriptional levels of the *mib1* gene cluster were significantly reduced in both mutants compared with wild-type ST4 (the bottom panel of Fig. 4C); therefore, we named them as *mibR1* (mating inhibitor biosynthesis regulator 1) and *mibR2* (mating inhibitor biosynthesis regulator 2), respectively (Fig. 4A). As expected, the *mibR1* and *mibR2* deletion mutants lost the ability to suppress the sexual mating of *S. scitanineum*, while the complemented strains restored their biocontrol ability to the wild-type level (the top panel of Fig. 4C).

These two regulators share about 50% similarity at the amino acid level. To assess whether MibR1 and MibR2 may form a heterologous protein complex, similar to the case of HrpR and HrpS as reported in *P. syringae* (37), we performed a bacterial two-hybrid (B2H) assay. As shown in Fig. S5, MibR1 and MibR2 did not interact with each other, suggesting that they may act independently in regulating the biocontrol activity of strain ST4.

### MibR1 and MibR2 act downstream of GacA in regulating the expression of the *mib1* gene cluster

**Fig 5:**
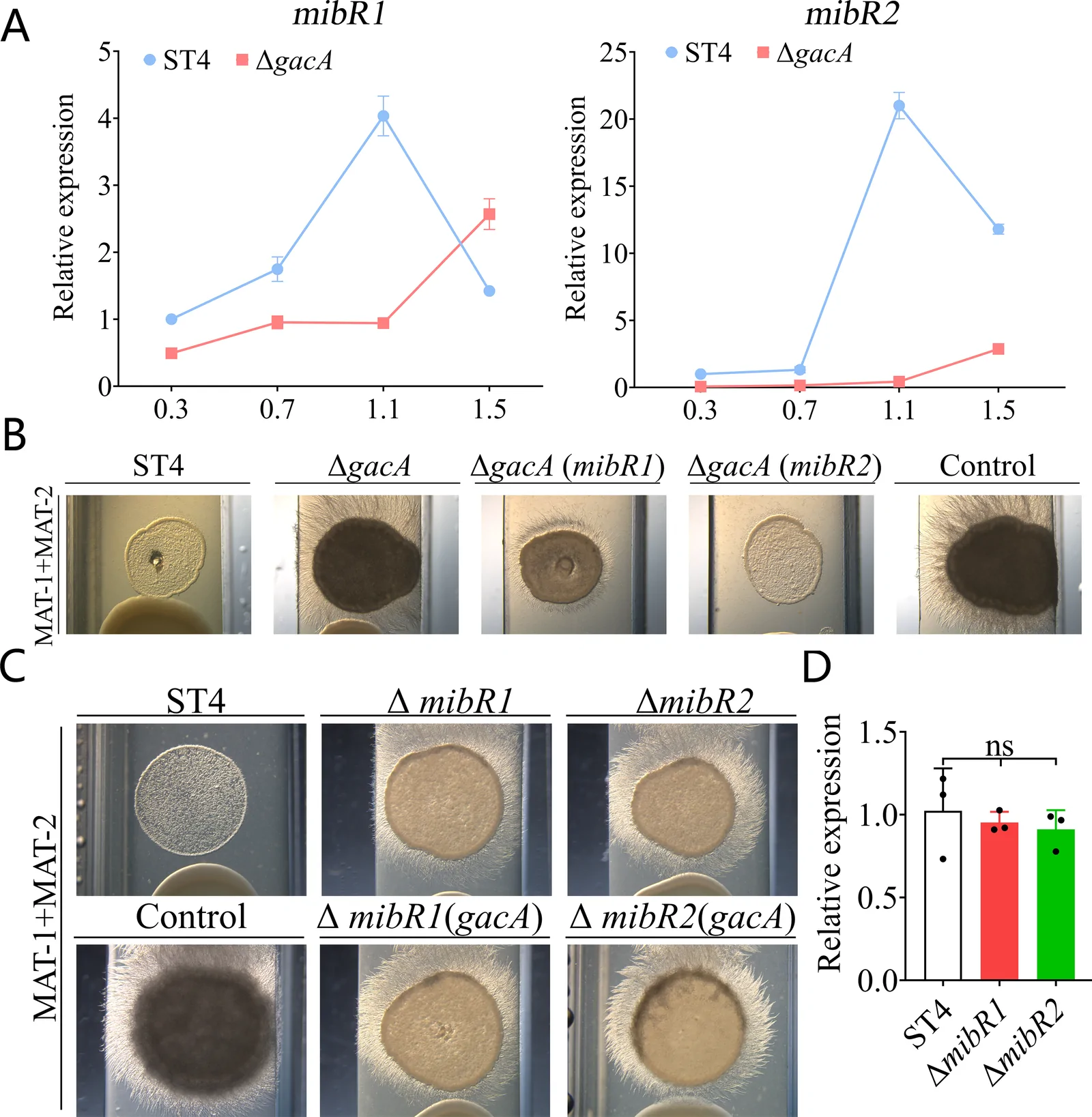
Transcriptional expression of *mibR1* and *mibR2* is regulated by GacA. (**A**) The *mibR1* and *mibR2* expression profiles in strain ST4 and the mutant Δ*gacA*. The *x*-axis represents OD_600_ values. (**B and C**) The colony morphology of *S. scitamineum* next to strain ST4 or its derivatives. Control: the fungal cells alone without strain ST4 or its derivative. (**D**) Relative expression of *gacA* measured by RT-qPCR in strain ST4, mutants Δ*mibR1* and Δ*mibR2*. Statistical analysis was performed using one-way ANOVA with multiple comparisons. Symbol: ns, not significant (*P* ≥ 0.05). Error bars indicate the standard error of the mean (*n* = 3). The experiments were done independently at least three times, and the representative images are shown here.

To determine whether MibR1 and MibR2 are acting downstream of the GacS/GacA regulatory pathway to control the expression of the *mib1* gene cluster, we extracted RNA from wild-type ST4 and mutant Δ*gacA* at different growth phases and quantified the transcript levels of *mibR1* and *mibR2*. The results showed that the expression level of *mibR1* in the *gacA* mutant was lower than that in the wild type at OD_600_ ≈ 0.3, 0.7, and 1.1, but higher than that in the wild type at OD_600_ ≈1.5 (Fig. 5A, left panel), while the level of *mibR2* in the *gacA* mutant was lower than that in the wild type at all four growth time points tested (Fig. 5A, right panel). These results indicate that expression of *mibR1* and *mibR2* is regulated by GacA. We individually overexpressed *mibR1* and *mibR2* in the *gacA* mutant and examined whether they could rescue the defective inhibitory activity of mutant Δ*gacA* against the fungal sexual mating. The results showed that overexpression of *mibR2* in the Δ*gacA* restored the ability to inhibit sexual mating to the wild-type level, whereas overexpression of *mibR1* only partially rescued the defective anti-mating activity (Fig. 5B). To test whether *mibR1* and *mibR2* act upstream of GacA, we overexpressed *gacA* in both the *mibR1* and *mibR2* single-deletion mutants, and neither mutant regained the ability to inhibit the fungal sexual mating (Fig. 5C). Furthermore, there was no difference in the transcript level of *gacA* in the *mibR1* or *mibR2* mutant compared with that in wild-type ST4 (Fig. 5D). Based on the above results, we conclude that MibR1 and MibR2 act downstream of GacA.

### Regulatory mechanisms that govern the expression of *mib1ABCDEFG*

**Fig 6:**
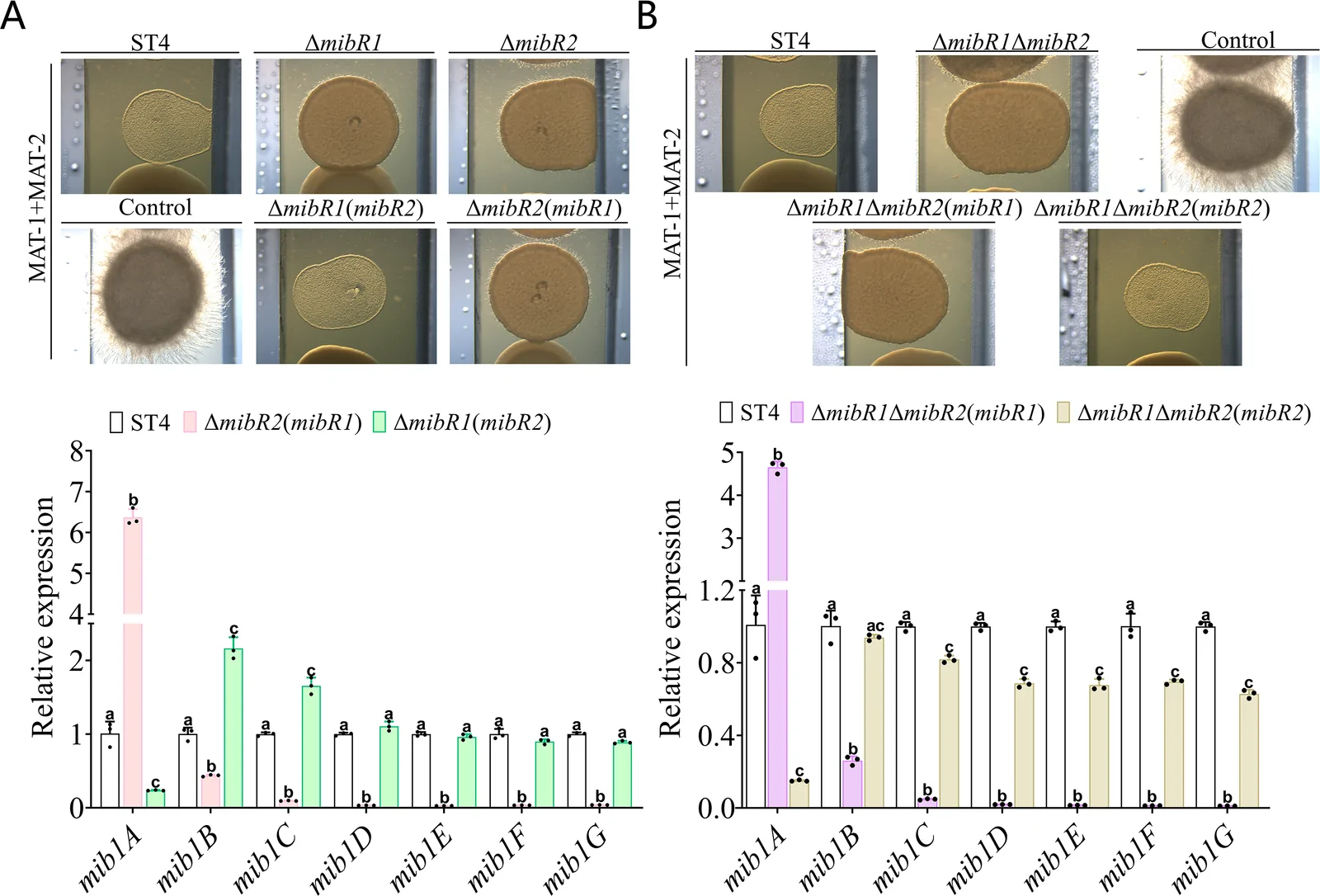
The relationship of MibR1 and MibR2 in the regulation of biocontrol phenotype and the *mib1* gene cluster expression. (**A**) The top panel shows the colony morphology of *S. scitamineum* next to strain ST4. Control: the fungal cells alone without strain ST4 or its derivative. The bottom panel shows relative expression of the *mib1* genes measured by RT-qPCR in strains ST4, Δ*mibR2*(*mibR1*), and Δ*mibR1*(*mibR2*). (**B**) The top panel shows the colony morphology of *S. scitamineum* next to strain ST4. Control: the fungal cells alone without strain ST4 or its derivative. The bottom panel shows relative expression of the *mib1* genes measured by RT-qPCR in strains ST4, Δ*mibR1*Δ*mibR2*(*mibR1*), and Δ*mibR1*Δ*mibR2*(*mibR2*). Strains Δ*mibR2*(*mibR1*), Δ*mibR1*(*mibR2*), Δ*mibR1*Δ*mibR2*(*mibR1*), and Δ*mibR1*Δ*mibR2*(*mibR2*) were constructed by overexpression of *mibR1* or *mibR2* in the corresponding single or double deletion mutant, respectively. Statistical analysis was performed using two-way ANOVA with multiple comparisons. The significance with respect to strain ST4 and the same letters indicate no significant difference, and different letters indicate significant differences (*P* < 0.05). Error bars indicate the standard error of the mean (*n* = 3). The experiments were done independently at least three times, and the representative images are shown here.

To determine the epistatic relationship between MibR1 and MibR2 in regulating the *mib1* gene cluster, we overexpressed *mibR2* in the *mibR1* mutant background and vice versa, and then respectively assessed the ability of the two resultant strains to inhibit the sexual mating of *S. scitamineum*. The results showed that overexpression of *mibR2* in the *mibR1* mutant restored the ability to inhibit sexual mating to the wild-type ST4 level, whereas overexpression of *mibR1* in the *mibR2* mutant failed to restore the ability to inhibit sexual mating (Fig. 6A, top panel). We also measured the expression levels of the *mib1* genes in these two strains. The results showed that in the *mibR1* mutant overexpressing *mibR2*, the transcript levels of *mib1B* and *mib1C* exceeded those in the wild type, while the transcript levels of the genes *mib1D*, *mib1E*, *mib1F*, and *mib1G* were restored to the wild-type level. In the *mibR2* mutant overexpressing *mibR1*, the expression level of *mib1A* was higher than that in the wild type, whereas the expression levels of *mib1B*, *mib1C*, *mib1D*, *mib1E*, *mib1F*, and *mib1G* remained lower than those in the wild type (Fig. 6A, bottom panel). To determine the relative roles of MibR1 and MibR2 in regulation of the *mib1* gene cluster, we generated a double-deletion mutant Δ*mibR1*Δ*mibR2* in the wild-type background and then individually complemented with *mibR1* or *mibR2*. Complementation with *mibR2* alone fully restored mating-inhibitory activity, whereas complementation with *mibR1* failed to do so (Fig. 6B, top panel). RT-qPCR analysis showed that in the mutant Δ*mibR1*Δ*mibR2* complemented with *mibR1*, the transcript level of *mib1A* exceeded that of the wild type, whereas the transcript level of genes *mib1B*, *mib1C*, *mib1D, mib1E*, *mib1F*, and *mib1G* was significantly lower than those in the wild type. In the double mutant Δ*mibR1*Δ*mibR2* complemented with *mibR2*, the transcript level of *mib1B* did not differ from that of the wild type, whereas expression of *mib1A* and *mib1C*, *mib1D, mib1E*, *mib1F*, and *mib1G* remained significantly lower than in the wild type (Fig. 6B, bottom panel). Nevertheless, compared with the mutant Δ*mibR1*Δ*mibR2* complemented with *mibR1*, the transcriptional levels of *mib1B*, *mib1C*, *mib1D, mib1E*, *mib1F*, and *mib1G* were markedly upregulated. Collectively, our results place MibR2 downstream of MibR1 in the regulatory pathway on the *mib1* gene cluster.

### GacS/GacA might control the expression of the *mibR1/mibR2-mib1* cluster axis through the conserved small RNA pathway

**Fig 7:**
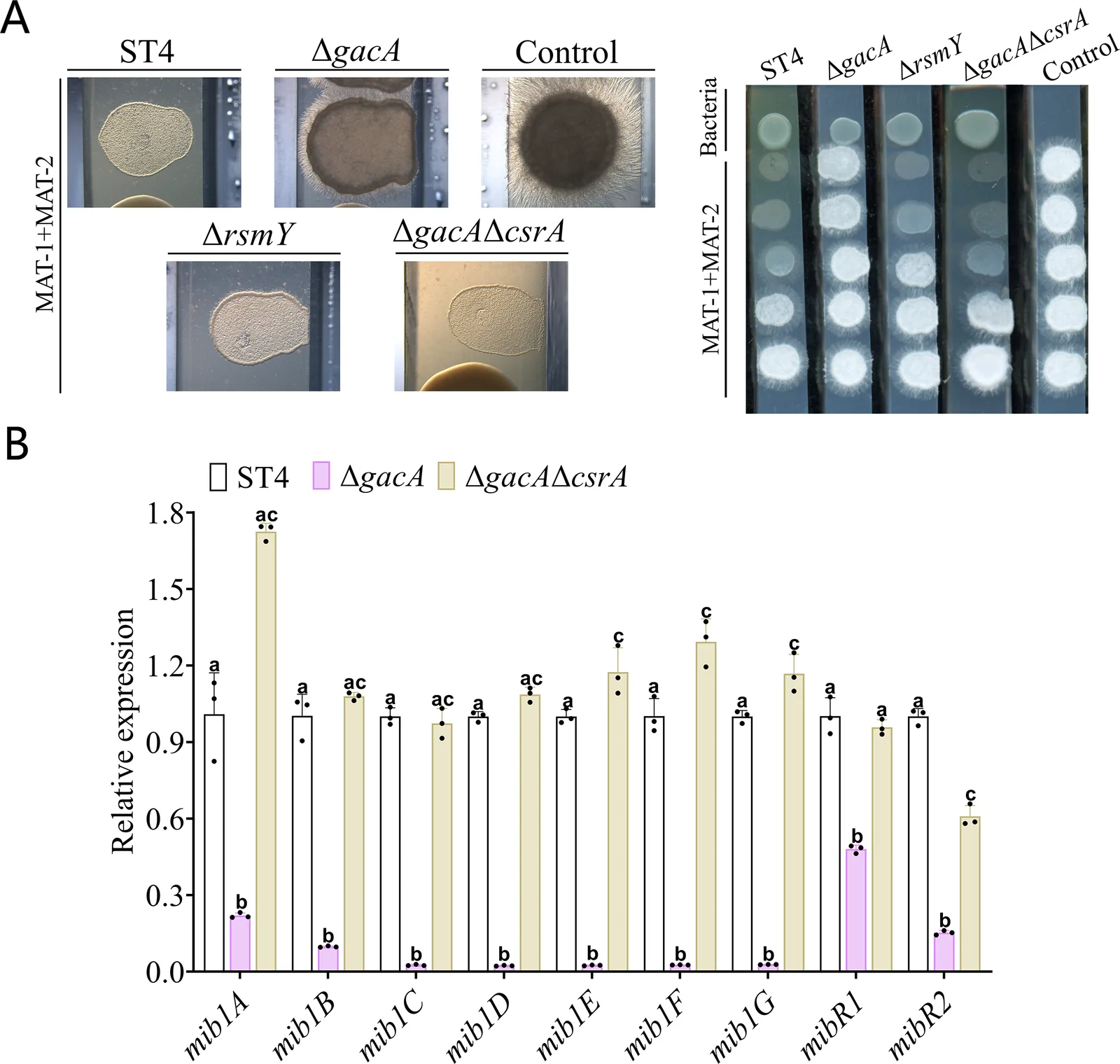
The roles of small RNA rsmY and the RNA binding protein CsrA in regulation of the anti-mating activity of strain ST4. (**A**) The left panel shows the colony morphology of *S. scitamineum* next to strain ST4 or its derivatives. The bottom panel shows the effect of *rsmY* or *csrA* deletion in the mutant Δ*gacA* background on the anti-mating activity of strain ST4. Control: the fungal cells alone without strain ST4 or its derivative. (**B**) Relative expression levels of the *mib1* genes measured by RT-qPCR in strain ST4, mutants Δ*gacA* and Δ*gacA*Δ*csrA*. Statistical analysis was performed using two-way ANOVA with multiple comparisons. Different letters above the bars indicate significant differences (*P* < 0.05). Error bars indicate the standard error of the mean (*n* = 3). The experiments were done independently at least three times, and the representative images are shown here.

The highly conserved Gac/Rsm signaling cascade has been reported (20), in which GacA promotes expression of the small RNA *rsmY* and *rsmZ*, which in turn sequester the translational repressor CsrA and thus relieve CsrA repression of the downstream transcriptional regulator(s). In the ST4 genome, only *rsmY* but not *rsmZ* was identified. We then constructed an *rsmY* deletion mutant. The bioassay results showed that deletion of *rsmY* reduced but did not abolish the ability to inhibit sexual mating (Fig. 7A). We then deleted *csrA* in the Δ*gacA* background to generate the double-deletion mutant Δ*gacA*Δ*csrA*. As expected, this mutant regained the inhibitory ability on the fungal sexual mating (Fig. 7A). Consistently, the transcriptional levels of the *mib1* genes in the mutant Δ*gacA*Δ*csrA* were fully restored to or even exceeded the wild-type levels (Fig. 7B). Concurrently, the transcription of *mibR1* in mutant Δ*gacA*Δ*csrA* was returned to the wild-type value, whereas the transcription of *mibR2* in mutant Δ*gacA*Δ*csrA* remained lower than that in the wild type, yet higher than that in the mutant Δ*gacA* (Fig. 7B). We therefore propose that the GacS/GacA system modulates the expression of the *mib1* cluster via sRNA RsmY and the cognate repressor CsrA.

## DISCUSSION

*S. scitamineum*, the causal agent of sugarcane smut disease, must undergo sexual mating before it can form dikaryotic hyphae to infect sugarcane. Our laboratory previously isolated *Pseudomonas shiyinii* strain ST4, which specifically inhibits the sexual mating and dikaryotic hyphal growth of *S. scitamineum* (14). To unveil this fascinating biocontrol mechanism, we screened for Tn5 transposon mutants of strain ST4 defective in inhibition of the fungal mating, which led to the identification of eight Tn5 disrupted genes putatively involved in mating inhibition. The predicted products of these genes belong to three categories, namely two-component system (*gacS/gacA*), pyrroloquinoline biosynthesis (*pqqBCDEF*), and a putative PQQ-dependent dehydrogenase (*gcd*), suggesting a GacS/GacA mediated PQQ-dependent enzymatic pathway in biosynthesis of the fungal mating inhibitor(s). Further characterization validated the key role of GacS/GacA and uncovered the *mib1* gene cluster as well as two transcriptional factors MibR1 and MibR2 acting hierarchically downstream of GacS/GacA, for biosynthesis of mating-inhibitory compound(s). Moreover, we showed that GacS/GacA modulates the expression of the *mib1* genes, *mibR1*, and *mibR2* through small RNA *rsmY* and the cognate repressor CsrA. These original findings outline the regulatory pathway essential for the biocontrol activity of strain ST4 and would facilitate further identification and characterization of its fungal mating inhibitor(s).

Given the dramatic effect of the *gacS/gacA* mutation on the mating inhibitory activity of strain ST4 (Fig. 1 and 2), transcriptomic analysis was conducted to identify the GacS/GacA modulated genes responsible for biosynthesis of the fungal mating inhibitor(s). Interestingly, among the Tn5 disrupted genes, the expression levels of the five genes encoding PQQ biosynthesis, *pqqBCDEF*, as well as the *gcd* gene, were not changed in the *gacS* and *gacA* deletion mutants (Table S6), suggesting that they are not under the control of the GacS/GacA two-component system. This speculation appears rational as PQQ is a common cofactor used by various enzymes (19).

The GacS and GacA of *Pseudomonas shiyinii* strain ST4 control a common set of genes but also seem to have separate regulons (Fig. 3A). This is not uncommon, as quite some sensor kinases and corresponding response regulators have individual regulons while sharing a common set of regulons (38, 39). In the case of the AmgS/AmgR two-component system of *P. aeruginosa* PAO1, a recent study showed that the sensor and the response regulator could regulate distinct sets of genes. Transcriptomic analysis showed that deletion of *amgS* resulted in upregulation of 233 genes and downregulation of 106 genes. In contrast, deletion of *amgR* led to the upregulation of 273 genes and downregulation of 83 genes. AmgS and AmgR share 118 commonly regulated genes, including 77 upregulated and 41 downregulated. Further study indicated that AmgR expression could be induced by certain antibiotics to upregulate multiple genes independent of its cognate sensor kinase AmgS (40). Obviously, the capability of the sensor and the response regulator to respond to the same or different environmental cues would increase the flexibility and functional spectrum of two-component systems in responding to environmental changes. It is interesting to note that, similar to *amgS* and *amgR*, the *gacS* and *gacA* in strain ST4 are located at separate genetic loci (Fig. S6). The molecular mechanism by which GacS and GacA regulate different sets of genes remains to be investigated.

To investigate the effect of the *mib1* gene cluster on inhibiting the sexual mating of *S. scitamineum*, we conducted gene deletion analysis, which demonstrated that, with the exception of the mutant Δ*mib1A*, which showed no effect on biocontrol function, deletion of the other six genes of the *mib1* gene cluster resulted in a significant reduction in the ability to inhibit sexual mating in *S. scitamineum*. Notably, the *mib1D* and *mib1G* deletion mutants almost completely lost the ability to inhibit sexual mating of *S. scitamineum,* similar to the *gacS* and *gacA* mutants, indicating that the enzymes encoded by *mib1D* and *mib1G* might catalyze the critical rate-limiting steps in the synthesis of sexual mating inhibitor(s) mediated by the *mib1* gene cluster, or alternatively, their products may also be used in the synthesis of other mating inhibitors. Based on the predicted functions of the proteins encoded by the genes within the *mib1* gene cluster and the known activities of their homologous proteins (Table S7), we propose that the anti-mating inhibitor synthesized by the *mib1* cluster is likely an amino acid-derived aromatic or heteroaromatic metabolite with a conjugated structure and likely possessing electrophilic and redox-active properties.

We performed a bioinformatic analysis of the *mib1* gene cluster to investigate its distribution among other bacteria (Fig. S3). Interestingly, the *mib1* gene clusters in *Cupriavidus gilardii* J11 and *Paraburkholderia antibiotica* strain G-4-1-8 lack the *mib1A* gene. In contrast, although the *mib1A* gene is present in *Burkholderia plantarii* strain ATCC 43733, its transcriptional orientation is opposite to that of the rest of the *mib1* gene homologs. Combined with the observation that deletion of the *mib1A* gene in strain ST4 had no effect on the ability of ST4 to inhibit the sexual mating of *S. scitamineum*, we hypothesize that the ancestral form of the *mib1* gene cluster consisted solely of the six core genes (*mib1B–mib1G*) and was likely originated from non-*Pseudomonas* genera such as *Cupriavidus* and *Paraburkholderia* via horizontal gene transfer (HGT). HGT between different bacterial genera is a common phenomenon. For instance, previous studies have reported that two *Burkholderia glumae* strains isolated from rice fields in Thailand (60BGCRMSO3-9 and 60BGCRMSO3-11) acquired an approximately 41.7 kb chromosomal island from the human pathogenic bacterium *Burkholderia pseudomallei* via HGT. This island contains genes encoding a PAAR domain-containing protein, a putative nuclease effector, and a type VI secretion system (T6SS) immunity protein, among others, with sequence identities to their homologs in *B. pseudomallei* reaching 90–100% (41).

Both MibR1 and MibR2 are members of the LysR-family transcriptional regulator family (Fig. S4). Numerous studies have shown that LysR-family transcriptional regulators are widely present in bacterial genomes, involved in regulating bacterial metabolism, toxin production, quorum sensing, and motility (42, 43). NmoR (NCBI ID: WP_447827183) and HinK (NCBI ID: AAG03607) from *P. aeruginosa* PAO1 are the closest homologs to MibR2, sharing about 40.28% and 42.03% similarities at the amino acid level, respectively. NmoR and HinK are known to regulate nitronate metabolism and biosynthesis of the quorum sensing signal PQS by directly binding to the corresponding target promoter, respectively (44, 45). The findings from this study clearly showed that MibR1 and MibR2 play critical roles as positive transcriptional factors modulating transcriptional expression of the *mib1* genes, as deleting either of them caused a drastic reduction in the transcript levels of the *mib1ABCDEFG* (Fig. 4C, bottom panel). Interestingly, overexpression of *mibR2* in the *mibR1* mutant could restore the fungal mating inhibitory activity to the wild-type ST4 level, but not vice versa (Fig. 6A, top panel). These findings indicate that MibR1 and MibR2 might work in tandem, with MibR1 functioning upstream and MibR2 acting downstream. It remains to be determined how these two transcription factors control the expression of the *mib1* gene cluster.

**Fig 8:**
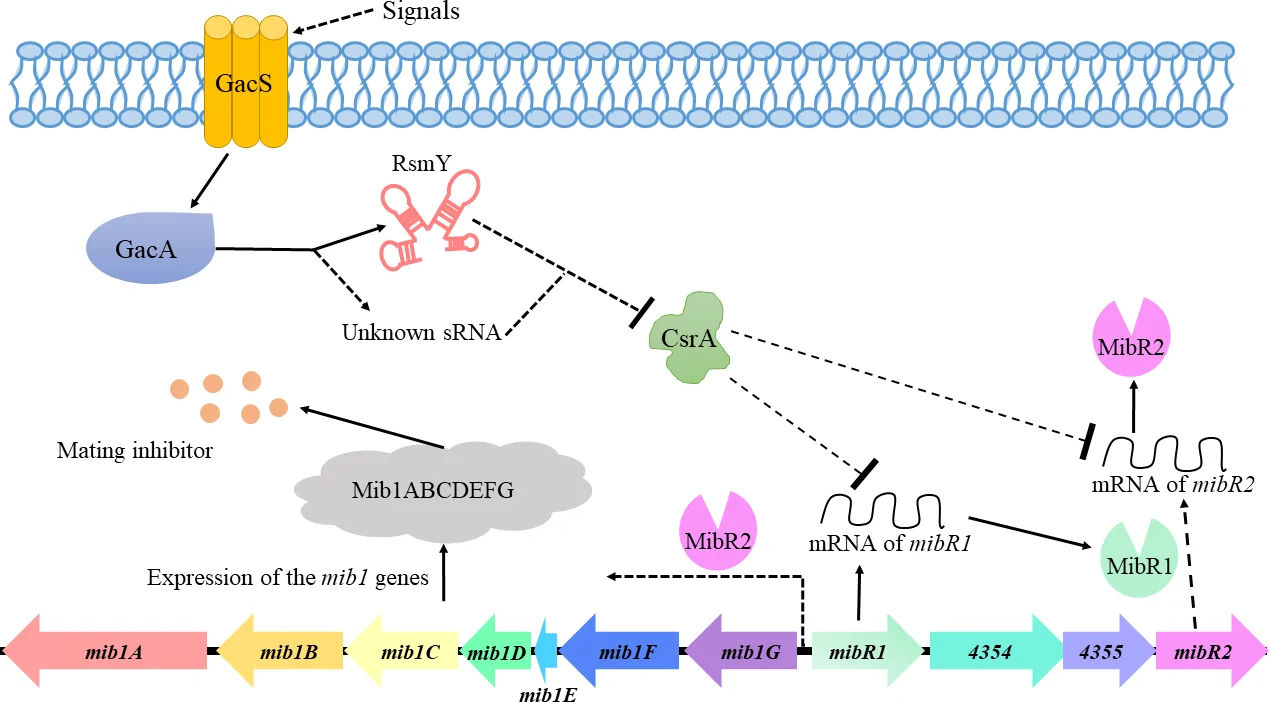
The regulatory network that modulates the anti-mating activity of *Pseudomonas shiyinii* ST4. Upon sensing unknown environmental cues, GacS activates its cognate response regulator GacA, which promotes the expression of the sRNA *rsmY* and likely another unknown sRNA. The sRNA molecules then sequester the translational repressor CsrA, allowing expression of the transcriptional regulator MibR1 and subsequently MibR2. MibR2 regulates the expression of the *mib1* gene cluster, thereby initiating the biosynthesis of the inhibitors against fungal sexual mating. The dashed line indicates that the identity of the mechanism awaits experimental verification.

Similar to its close relative *P. aeruginosa*, in which the GacS/GacA two-component system controls the transcriptional expression of downstream target genes through small RNA RsmY and RsmZ, and the RNA binding proteins CsrA and RsmF (20, 46), the GacS/GacA of ST4 regulates the *mib1* gene cluster through small RNA *rsmY* and its cognate small RNA binding protein CsrA (Fig. 7A). Interestingly, there is no *rsmZ* homolog in strain ST4, whereas evidence suggests the existence of a potential counterpart, as deletion of *rsmY* only partially attenuated the regulatory strength of the GacS/GacA system on the anti-mating activity of strain ST4 (Fig. 7A). It is also evident that the transcriptional regulators MibR1 and MibR2 work downstream of the Gac/Rsm pathway, as deletion of the repressor gene *csrA* in the genetic background of mutant Δ*gacA* substantially restored the transcript levels of *mibR1* and *mibR2* (Fig. 7B). In summary, we discovered a previously unknown biosynthetic gene cluster encoding anti-mating inhibitor(s) in the biocontrol agent *Pseudomonas shiyinii* ST4, which may be under the control of a regulatory cascade: GacS/GacA two-component system through a hierarchy of RsmY (sRNA)-CsrA (translational repressor)-MibR1/MibR2 (transcriptional regulators) (Fig. 8). These original findings enrich our understanding of the regulatory mechanisms that govern the biosynthesis of active biocontrol compounds by ST4 and lay the groundwork for screening and identification of such compound(s) synthesized by the *mib1* gene cluster.

## MATERIALS AND METHODS

### Bacterial strains, plasmids, primers, and culture conditions

The strains and plasmids used in this study are listed in Table 2. Primers used in this study are listed in Table S8. Strain ST4 and derivatives were routinely maintained at 28°C in Luria-Bertani (LB) medium. *Escherichia coli* strains were routinely maintained in LB medium at 37°C. The *S. scitamineum* haploid sporidia MAT-1 and MAT-2 were routinely maintained at 28°C in YePS medium (20 g peptone, 10 g yeast extract, and 10 g sucrose per L). PDA (200 g potato, 20 g glucose, and 18 g agar per L) was used for bioassay of bacterial antagonistic activity against sexual mating of S. *scitamineum. Pseudomonas* isolation agar (PIA, 20 g peptone, 1.4 g MgCl_2_, 10 g K_2_SO_4_, 25 mg Irgasan, and 15 g agar per L) and 10% LBwithout NaCl medium were used for generation of mutants and complemented strains. MM [minimal medium: K_2_HPO_4_ 10.5 g/L, KH_2_PO_4_ 4.5 g/L, (NH4)_2_SO_4_ 2.0 g/L, mannitol 2.0 g/L, glycerol 2.0 g/L, MgSO_4_·7H_2_O 0.2 g/L, CaCl_2_ 0.01 g/L, FeSO_4_·7H_2_O 0.005 g/L, and MnCl_2_·4H_2_O 0.002 g/L, pH 7.0, sterilization at 121°C for 20 min] was used for screening Tn5 mutants. The following antibiotics were supplemented when required: 50 mg/mL ampicillin, 50 mg/mL kanamycin, 50 mg/mL gentamicin, 15 mg/mL tetracycline, and 30 mg/mL chloramphenicol.

**TABLE 2:** Strains and plasmids used in this study

Strain or plasmid	Relevant characteristic(s)^*a*^	Source or reference
*Sporisorium scitamineum* strains		
MAT-1	Haploid sporidia of *S. scitamineum*	Laboratory collection
MAT-2	Haploid sporidia of *S. scitamineum*	Laboratory collection
*Pseudomonas shiyinii* strains		
ST4	Wild-type strain Amp^r^	Laboratory collection
Δ*gacS*	ST4 with the deletion of the *gacS* gene Amp^r^	This study
Δ*gacA*	ST4 with the deletion of the *gacA* gene Amp^r^	This study
Δ*gacS(gacS)*	Δ*gacS* containing the expression construct pTn7T-*gacS* Gen^r^	This study
Δ*gacA(gacA)*	Δ*gacA* containing the expression construct pTn7T-*gacA* Gen^r^	This study
Δ*4346*	ST4 with the deletion of the *4346* gene Amp^r^	This study
Δ*4347*	ST4 with the deletion of the *4347* gene Amp^r^	This study
Δ*4348*	ST4 with the deletion of the *4348* gene Amp^r^	This study
Δ*4349*	ST4 with the deletion of the *4349* gene Amp^r^	This study
Δ*4350*	ST4 with the deletion of the *4350* gene Amp^r^	This study
Δ*4351*	ST4 with the deletion of the *4351* gene Amp^r^	This study
Δ*4352*	ST4 with the deletion of the *4352* gene Amp^r^	This study
Δ*4353*	ST4 with the deletion of the *4353* gene Amp^r^	This study
Δ*4356*	ST4 with the deletion of the *4356* gene Amp^r^	This study
Δ*4347*(*4347*)	Δ*4347* containing the expression construct pTn7T-p*SsrA*_BCD_-*4347* Gen^r^	This study
Δ*4348*(*4348*)	Δ*4348* containing the expression construct pTn7T-p*SsrA*_BCD_-*4348* Gen^r^	This study
Δ*4349*(*4349*)	Δ*4349* containing the expression construct pTn7T-p*SsrA*_BCD_-*4349* Gen^r^	This study
Δ*4350*(*4350*)	Δ*4350* containing the expression construct pTn7T-p*SsrA*_BCD_-*4350* Gen^r^	This study
Δ*4351*(*4351*)	Δ*4351* containing the expression construct pTn7T-p*SsrA*_BCD_-*4351* Gen^r^	This study
Δ*4352*(*4352*)	Δ*4352* containing the expression construct pTn7T-p*SsrA*_BCD_-*4352* Gen^r^	This study
Δ*4353*(*4353*)	Δ*4353* containing the expression construct pTn7T-p*SsrA*_BCD_-*4353* Gen^r^	This study
Δ*4356*(*4356*)	Δ*4356* containing the expression construct pTn7T-p*SsrA*_BCD_-*4356* Gen^r^	This study
Δ*mibR1*(*gacA*)	Δ*4353* containing the expression construct pTn7T-*gacA* Gen^r^	This study
Δ*mibR2*(*gacA*)	Δ*4356* containing the expression construct pTn7T-*gacA* Gen^r^	This study
Δ*mibR1*Δ*mibR2*	ST4 with the deletion of the *4353* gene and *4356* gene Amp^r^	This study
Δ*mibR1*Δ*mibR2*(*mibR1*)	Δ*4353*Δ*4356* containing the expression construct pTn7T-p*SsrA*_BCD_-*4353* Gen^r^	This study
Δ*mibR1*Δ*mibR2*(*mibR2*)	Δ*4353*Δ*4356* containing the expression construct pTn7T-p*SsrA*_BCD_-*4356* Gen^r^	This study
Δ*mibR1* (*mibR2*)	Δ*4353* containing the expression construct pTn7T-p*SsrA*_BCD_-*4356* Gen^r^	This study
Δ*mibR2*(*mibR1*)	Δ*4356* containing the expression construct pTn7T-p*SsrA*_BCD_-*4353* Gen^r^	This study
Δ*gacA*(*mibR1*)	Δ*gacA* containing the expression construct pTn7T-p*SsrA*_BCD_-*4353* Gen^r^	This study
Δ*gacA*(*mibR2*)	Δ*gacA* containing the expression construct pTn7T-p*SsrA*_BCD_-*4356* Gen^r^	This study
Δ*gacA*Δ*csrA*	ST4 with the deletion of the *gacA* gene and *csrA* gene Amp^r^	This study
Δ*rsmY*	ST4 with the deletion of the *rsmY* gene Amp^r^	This study
*E. coli* strains		
DH5α	F-φ80*lacZ*ΔM15Δ(*lacZYA-argF*)*U169*, *endA1 recA1 hsdR17*(r_k_- m_k_+) *supE*44 λ- *thi-1 gyrA96 relA1 phoA*	TransGen Biotech, China
DH5α(pTNS2)	Helper strain encoding *tnsABCD*, Amp^r^	Laboratory collection
HB101(pRK2013)	*thr leu thi recA hsdR hsdM pro*, Kan^r^	Laboratory collection
Plasmids		
pUC18T-mini-Tn7T-Gm	*aacC1* on miniTn7-based vector with transcriptional terminators at the right end of the Tn7 transposon, Gen^r^	Laboratory collection
pTn7T-*gacA*	mini-Tn7 harboring the fragment of *gacA*, Gen^r^	This study
pTn7T-*gacS*	mini-Tn7 harboring the fragment of *gacS*, Gen^r^	This study
pTn7T-p*SsrA*_BCD_-*4353*	*4353* fused to the upstream region of gene *ssrA* in ST4, the BCD2 element, Gen^r^	This study
pTn7T-p*SsrA*_BCD_-*4356*	*4356* fused to the upstream region of gene *ssrA* in ST4, the BCD2 element, Gen^r^	This study
pTn7T-p*SsrA*_BCD_-*4347*	*4347* fused to the upstream region of gene *ssrA* in ST4, the BCD2 element, Gen^r^	This study
pTn7T-p*SsrA*_BCD_-*4348*	*4348* fused to the upstream region of gene *ssrA* in ST4, the BCD2 element, Gen^r^	This study
pTn7T-p*SsrA*_BCD_-*4349*	*4349* fused to the upstream region of gene *ssrA* in ST4, the BCD2 element, Gen^r^	This study
pTn7T-p*SsrA*_BCD_-*4350*	*4350* fused to the upstream region of gene *ssrA* in ST4, the BCD2 element, Gen^r^	This study
pTn7T-p*SsrA*_BCD_-*4351*	*4351* fused to the upstream region of gene *ssrA* in ST4, the BCD2 element, Gen^r^	This study
pTn7T-p*SsrA*_BCD_-*4352*	*4352* fused to the upstream region of gene *ssrA* in ST4, the BCD2 element, Gen^r^	This study
pK18mobsacB	Broad-host-range sucrose counterselection allelic exchange vector, *sacB*, Gm^r^	Laboratory collection
pTRG-*mibR2*	pTRG harboring the fragment of *4356*, Tc^r^	This study
pBT-*mibR1*	pBT harboring the fragment of *4353*, Cm^r^	This study

^*a*^ Amp^r^, ampicillin resistance; Kan^r^, kanamycin resistance; Gen^r^, gentamicin resistance; Tc^r^, tetracycline resistance; Cm^r^, chloramphenicol resistance.

### Random transposon mutagenesis

The overnight culture of *E. coli* S17-1 (containing plasmid pTGN) and the wild-type ST4 from solid LB medium plate were resuspended with 1 mL of MM, and adjusted to the same OD_600_ at about 1.5–2.0. Bacterial cell cultures of these two strains were mixed together in equal proportion with gentle vortexing. The mixture was then spotted as discrete droplets onto an LB plate and incubated at 28°C for 6 h. After incubation, bacterial cells were washed off the plates using a 2 mL MM liquid medium and spread evenly on the selective MM plates containing 50 μg/mL gentamicin and ampicillin, followed by incubation at 28°C for 24 h. The colonies appearing on the selective plates were separately spread on fresh selective plates for further purification. The purified colonies were inoculated respectively in LB liquid medium, cultured at 28°C overnight, and used for anti-mating bioassay as described below.

### Bioassay on inhibition of *S. scitamineum* sexual mating

The anti-mating bioassay was based on the established method with minor modifications (14). Briefly, PDA plates were cut into separated slices (0.7 cm in width). An aliquot of 1.3 μL bacterial culture (OD_600_ ≈ 1.0) was added to one end of an agar slice. Equal volumes of *S. scitamineum* haploid cell MAT-1 and MAT-2 cultures at OD_600_ ≈ 1.5 were taken and mixed at a 1:1 ratio. An aliquot of 1.3 μL of the fungal mixture was separately spotted per 0.7 cm on the slice from the loaded bacterial sample. LB medium was added in the same way as the bacterial culture as a negative control. The plates were incubated at 28°C for 2 days prior to phenotype observation and photographing. The Tn5 mutants with altered anti-mating activity were further verified by repeating the anti-mating bioassay, and the verified colonies were prepared as LB culture stocks containing 20% glycerol and kept at −80°C until further use.

### High-efficiency thermal asymmetric interlaced PCR

Based on the known Tn5 transposon sequence, three downstream border-specific primers (G1–G3), four random-degenerate primers (LAD1–LAD4), and specific primer AC1 were synthesized following the protocols described previously (47). All the primer sequences used in this study are listed in Table S8. The third-round PCR products were purified using the Gel Extraction kit (Omega, USA), then gently mixed with 1 μL of pEASY-T5 Zero Cloning Vector (TransGen Biotech, China) and 4 μL of the purified PCR products and incubated at 25°C for 30 min. The ligated T-vector was heat-shock-transformed into Trans1-T1 competent cells. Transformants with insert sizes matching the third-round PCR product were selected for sequencing analysis. After trimming the T-cloning vector and Tn5 transposon sequences, the flanking sequences of Tn5 insertion were obtained, which were blasted against the strain ST4 genome (NCBI GenBank accession number JBWZCP000000000.1) to retrieve the full-length sequences of Tn5-inserted genes.

### Construction of strain ST4 in-frame deletion mutants and complemented strains

A SacB-based approach was employed to achieve gene deletion in strain ST4 according to a previously described method (48). Briefly, a 300 bp upstream and a 300 bp downstream sequence of the target gene were amplified by PCR with Phanta Max Super-Fidelity DNA polymerase (Vazyme, China). The PCR products were assembled into the suicide plasmid pK18mobsacB that was predigested with BamHI and HindIII. The resultant constructs were introduced into strain ST4 using the helper plasmid pRK2013 by triparental mating. Deletion mutants were counter-selected using LB medium containing 10% sucrose and verified by PCR and DNA sequencing.

For gene complementation or overexpression, a 501 bp upstream flank from the *ssrA* gene of strain ST4, the translation coupler BCD2 (Table S8), and the open reading frames (ORFs) of the target gene were amplified by PCR and ligated with the linear vector pTn7T (digested with BamHI and HindIII) using the One Step Cloning kit. The resultant constructs were introduced into *Pseudomonas shiyinii* ST4 or mutants as indicated using the helper strain HB101 (pRK2013) and DH5α (pTNS2) to generate complementation or overexpression strains (49). The resultant strains were verified by PCR and subsequent DNA sequencing.

### RNA extraction and quantification of transcription

Bacterial cells were grown in PDB medium in the absence of fungal MAT-1 and MAT-2 cells and harvested at an OD_600_ of 1.0 by centrifugation. The Eastep super total RNA extraction kit (Promega, USA) was used for RNA purification. After electrophoretic quality examination, the RNA samples with two replicates were sent to Gene Denovo (China) for transcriptome sequencing analysis. RT-qPCR validation of transcriptome data and quantification of gene expression levels were performed as follows: RNA samples were prepared according to the method described above, the RNA concentration was measured, and 1 μg of RNA was used to generate cDNA using the HiScript II Q RT SuperMix for qPCR (+gDNA wiper) (Vazyme, China). Quantitative real-time PCR (RT-qPCR) was performed in the QuantStudio 6 real-time detection system (Applied Biosystems, USA) using the ChamQ Universal SYBR qPCR master mix (Vazyme). The RNA polymerase sigma factor gene *rpoD* was selected as an internal control. The relative gene expression level was calculated by using the 2^−ΔΔCT^ method. The result was displayed as the mean of three biological repeats and four technical replicates.

### Bacterial two-hybrid assay

The B2H assay was conducted using the BacterioMatch II Two-Hybrid System Vector Kit following the manufacturer’s protocol (Agilent Technologies, USA). Approximately 100 ng of each plasmid harboring pTRG-*mibR1* and pBT-*mibR2* were co-transformed into the host strain XL1-Blue MRF’ Kan through chemical transformation after adding 500 μL LB medium to each tube. The cells were then incubated at 37°C with shaking at 200 rpm for 2 h. PCR and DNA sequencing were conducted to select the successfully co-transformed colonies on LB medium containing tetracycline (15 μg/mL) and chloramphenicol (30 μg/mL) for subsequent experiments. Thereafter, co-transformed colonies were cultured in liquid LB medium to OD_600_ = 1.0, then resuspended in 1 mL sterile water and then grown on nonselective (0 mM 3-AT) and selective (0.5 mM 3-AT) screening medium for 24–36 h at 37°C.

### RT-PCR analysis of the *mib1* (*4346–4352*) operon

This assay is used to verify whether the genes *4346*, *4347*, *4348*, *4349*, *4350*, *4351*, and *4352* share the same promoter. The cDNA sample was generated following the method described in the “RNA extraction and quantification of transcription” section. Using cDNA as the template, Green Taq (Vazyme) was employed for PCR analysis with the primers listed in Table S8. The PCR products were analyzed by agarose gel electrophoresis to determine their sizes.

### Statistical analysis

All the experiments were individually performed at least three times in three replicates each time. Data shown are the means from three replicates, and error bars indicate the standard deviations or standard errors. Statistical comparison was performed by using two-way ANOVA and one-way ANOVA with multiple comparisons in GraphPad Prism 8.0 software (GraphPad, La Jolla, CA, USA).

## Data Availability

The genome sequence of *Pseudomonas shiyinii* strain ST4 is available in NCBI GenBank under accession JBWZCP000000000.1. The raw RNA-seq data of *Pseudomonas shiyinii* strain ST4 and its mutants are accessible in the NCBI database under BioProject accession number PRJNA1451954. The constructed strains and plasmids from this study can be obtained by contacting the last corresponding author (dengyz@scau.edu.cn).

## SUPPLEMENTAL MATERIAL

Supplemental figures aem.00321-26-s0001.docx Fig. S1 to S6.

Supplemental tables aem.00321-26-s0002.xlsx Tables S1 to S8.

ASM does not own the copyrights to Supplemental Material that may be linked to, or accessed through, an article. The authors have granted ASM a non-exclusive, world-wide license to publish the Supplemental Material files. Please contact the corresponding author directly for reuse.
