# Supplementary material for: Pseudomonas shiyinii ST4 inhibits sexual mating of Sporisorium scitamineum through the GacS/GacA two-component system and the mib1 gene cluster
Source: Appl Environ Microbiol. 2026 Sep 15;92(10):e00321-26. doi: 10.1128/aem.00321-26 (PMC13689391; doi:10.1128/aem.00321-26)
Supplement: Supplementary file 1 [file aem.00321-26-s0001.docx]

**Supplemental Material**

***Pseudomonas shiyinii* ST4 inhibits sexual mating of *Sporisorium scitamineum* through the GacS/GacA two-component system and the *mib1* gene cluster**

Yihang Yang^a#^, Nuoqiao Lin^a, b#^, Jiahui Huang^a^, Weihan Gu^a^, Zhijian Hu^a^, Kai Yin^c^, Xinping Bi^a^, Ling He^a^, Lian-Hui Zhang^a*^, Zhibin Liang ^a*^, Yizhen Deng ^a^*

^a^Guangdong Provincial Key Laboratory of Microbial Signals and Disease Control, Integrative Microbiology Research Center, South China Agricultural University, Guangzhou 510642, China

^b^State Key Laboratory of Biocontrol, School of Life Sciences, Sun Yat-Sen University, Guangzhou 510275, China

^c^Hubei Key Laboratory of Edible Wild Plants Conservation and Utilization, College of Life Sciences, Hubei Normal University, Huangshi 435002, China

*Correspondence:	Lian-Hui Zhang, [lhzhang01@scau.edu.cn](mailto:lhzhang01@scau.edu.cn) or

Zhibin Liang, liangzb_gz@scau.edu.cn or

Yizhen Deng, dengyz@scau.edu.cn

^#^Contributed equally to this work.

**FIG S1** The genetic map of gene clusters positively regulated by *gacS* and *gacA*. Red and black numbers respectively represent the gene log2FC values of the *gacS* and *gacA* mutants in comparison with WT strain ST4. The positive values and negative values indicate the genes being up-regulated and down-regulated upon mutation, respectively.

**FIG S2** RT-PCR verification of the *mib1ABCDEFG* (*4346*–*4352*) operon.

**FIG S3** Conservation of the *mib1* gene cluster in other bacterial species. Genetic organization of the *mib1* gene cluster in *Pseudomonas*, *Burkholderia*, *Cupriavidus*, and *Paraburkholderia* species. The numbers indicate the sequence similarity at amino acid level (identity at nucleotide level) of each gene compared to the corresponding gene in the *mib1* gene cluster of *Pseudomonas shiyinii* ST4.

**FIG S4** The domain structures of MibR1 (4353) (A) and MibR2 (4356) (B).

**FIG S5** Results of the bacterial two-hybrid assay of MibR1 and MibR2. All experiments were performed independently at least three times, and representative images are shown here.

**FIG S6** The genetic loci of *gacS* and *gacA* in the genome of strain ST4.
